# Supplementary material for: Development of a computer-aided design software for the quantitative evaluation of aesthetic damage
Source: PLoS One. 2019 Dec 18;14(12):e0226322. doi: 10.1371/journal.pone.0226322 (PMC6919621; doi:10.1371/journal.pone.0226322)
Supplement: S1 Code site — 1. (ZIP) [file pone.0226322.s001.zip › [EESC_jr][Projeto]Relatorio_07_21/vendors/select2/docs/_includes/nav/options.html]

- Core options
  - Changing default options
  - Declaring configuration in the HTML
  - AMD compatibility
- Data adapters
  - Using a `<select>`
  - Loading data from an array
  - Connecting to a remote data source
- Displaying selections
  - Showing a placeholder
  - Clearing selections
  - Templating
- Displaying results
  - Controlling result loading
  - Making selections
  - Placement
- Events
  - Public jQuery events
  - Internal events
- The plugin system (adapters)
  - All adapters
  - Container (selection)
  - Data set
  - Dropdown
  - Results
- Deprecated options
  - Simplified function for matching data objects
  - Old initial selections with `initSelection`
  - Querying old data with `query`
  - Compatibility with `<input type="text" />`

Back to top
